# Treatment of Scarlet Fever and Diphtheria by Diniodide of Mercury

**Published:** 1891-10-03

**Authors:** C. R. Illingworth

**Affiliations:** Clayton-le-Moors


### Editor's Letter-Box.

[Our correspondents are reminded that prolixity is a great bar to publication, and that brevity of style and conciseness of statement greatly facilitate early insertion].

### THE TREATMENT OF SCARLET FEVER AND DIPHTHERIA BY THE BINIODIDE OF MERCURY.

Sir,—Permit me to quote one or two opinions upon this treatment and to make one or two remarks. "I have now given the Hg. $I_2$ in several cases of scarlet fever, with this result: that it not only arrests the fever, but it prevents the desquamation of the skin, or arrests it to such an extent that only slight scurfiness of the skin of the hands and feet arises." (Dr. Clement Dukes, Physician to the Rugby School. *B.M.J.*, July 9, 1887). "In a case of diphtheria, temperature 140 deg. with very rapid pulse, and the fauces covered with false membrane, I prescribed the biniodide every two hours. The next day the temperature was 100 deg., the pulse 90; the throat was clearing, and swelling of glands subsiding. The father of the patient was much struck with the improvement. In other forty-eight hours the temperature was subnormal and the exudation gone. I then gave iron and chlorate of potash" (Dr. H. Gabb, *B.M.J.*, July 21, 1888). "I have successfully treated about two hundred cases of scarlet fever and a few cases of puerperal fever by the biniodide" (Dr. Sexton, Kilrush, County Clare, Ireland). "Dr. Curdy treated 50 cases by biniodide and believed it hastened a fall in the temperature, lessened the chance of lingering sequelæ, and diminished the mortality. Dr. Mayo also thought the treatment beneficial in some cases; but Dr. Jacob, Mr. Rumboll, Dr. Swann, and Mr. Hick who had all tried the method, were not in favour of it, and it was thought by Dr. Hick that the tendency to nephritis was rather increased by pursuing it. (*B.M.J.*, January 19, 1889.) "This method has been tried at the London Fever Hospital without beneficial results" (Dr. Sydney Phillips, "Year Book of Treatment," 1890, p. 140). "The salicylate of soda is valueless in the graver forms of diphtheria, and especially when there is exudation either in the nostrils or larynx. In these cases biniodide of mercury with bark is the remedy I now employ" (Dr. Lennox Browne, *B.M.J.*, August 1, 1891).

Now, since May, 1886, on the first day of which month I published the discovery I had made regarding the potency of this drug against the scarlatinal and diphtheritic poisons, I have over and over again proved to my own satisfaction and that of others who have watched my practice, that, used as I have directed, it promptly arrests these fearful disorders. In the case of scarlet fever the defervescence begins on the first day, and the rash has disappeared by the fifth instead of being at its height. I have also proved that when given as I directed, and used as I prescribed, nephritis in no single instance has followed.—*Vide* "Treatment by Biniodide of Mercury." (H. K. Lewis, London.)

In conclusion, therefore, I beg to submit that Doctors Jacob, Rumboll, Swann, Hick, and Phillips, have erred in their methods of administering and applying the remedy, and this most probably from insufficient knowledge of my method, because none of them ever communicated with me upon the subject, and it was only a few months ago that I became aware of the fact of the treatment having received the favour of a trial in the London Fever Hospital.—I am, Sir, yours faithfully, C. R. Illingworth, M.D.

Clayton-le-Moors, September 7th, 1891.

